# Easy-to-Fabricate and High-Sensitivity LSPR Type Specific Protein Detection Sensor Using AAO Nano-Pore Size Control

**DOI:** 10.3390/s17040856

**Published:** 2017-04-13

**Authors:** Sae-Wan Kim, Jae-Sung Lee, Sang-Won Lee, Byoung-Ho Kang, Jin-Beom Kwon, Ok-Sik Kim, Ju-Seong Kim, Eung-Soo Kim, Dae-Hyuk Kwon, Shin-Won Kang

**Affiliations:** 1 School of Electronics Engineering, College of IT Engineering, Kyungpook National University, 1370 Sankyuk-dong, Bukgu, Daegu 702-701, Korea; kei95304@gamil.com (S.-W.K.); jslee_1245@naver.com (J.-S.L.); sw3148@ee.knu.ac.kr (S.-W.L.); jinbumkwon@naver.com (J.-B.K.); oskim@knu.ac.kr (O.-S.K.); jskim5772@ee.knu.ac.kr (J.-S.K.); 2 Division of Advanced Research and Development, SINOKOR, 12 Seongseogongdanbuk-ro 43-gil, Dalseo-gu, Daegu 704-920, Korea; bhkang@ee.knu.ac.kr; 3 Division of Computer and Electronic Engineering, Pusan University of Foreign studies, 65 Namsan-dong, Geumjeong-gu, Busan 608-738, Korea; eskim@pufs.ac.kr; 4 Department of Electronics Engineering, Kyungil University, Hayang-up, Gyeongsang buk-do 712-702, Korea; dhkwon@kiu.ac.kr

**Keywords:** localized surface plasmon resonance (LSPR), optical interferometry, anodic aluminum oxide (AAO), pore-size, pore-area, serum amyloid A1 (SAA1), optical-biosensor, C-reactive protein (CRP)

## Abstract

In this study, we developed a pore size/pore area-controlled optical biosensor-based anodic aluminum oxide (AAO) nanostructure. As the pore size of AAO increases, the unit cell of AAO increases, which also increases the non-pore area to which the antibody binds. The increase in the number of antibodies immobilized on the surface of the AAO enables effective detection of trace amounts of antigen, because increased antigen-antibody bonding results in a larger surface refractive index change. High sensitivity was thus achieved through amplification of the interference wave of two vertically-incident reflected waves through the localized surface plasmon resonance phenomenon. The sensitivity of the fabricated sensor was evaluated by measuring the change in wavelength with the change in the refractive index of the device surface, and sensitivity was increased with increasing pore-size and non-pore area. The sensitivity of the fabricated sensor was improved and up to 11.8 ag/mL serum amyloid A1 antigen was detected. In addition, the selectivity of the fabricated sensor was confirmed through a reaction with a heterogeneous substance, C-reactive protein antigen. By using hard anodization during fabrication of the AAO, the fabrication time of the device was reduced and the AAO chip was fabricated quickly and easily.

## 1. Introduction

Biosensors based on nanoscience have been developed to improve the sensitivity compared to previous bulk-state-based biosensors. In addition, these biosensors exhibit the characteristic advantages of the functional nano-size structures and devices from the early 1970s [1,2,3,4]. Typically, nano-structures may be in any of the following forms: nanodots, nanowires, nanorods, nanotubes, etc. Nano-structure-based biosensors show high sensitivity and resolution because of their large binding number of sites resulting from larger surface area and high aspect ratio.

In the past few decades, optical-based biosensors and semiconductor-based biosensors have been developed. Several methods have been developed to diagnose diseases using optical biosensors. Chen et al. [5] and Alexandre et al. [6] reported surface plasmon resonance (SPR)-based biosensors that function through physical adsorption. This method exhibits high resolution and sensitivity; however, it is unsuitable for immunosensing because physical adsorption is required. In addition, several groups developed waveguide biosensors that measure changes in the refractive index and interferometry, as introduced by Gao et al. and Ligler et al. [7,8]. However, these sensors have a low limit of detection (~30–50 pg/mL). For semiconductor-based biosensors, Yuan et al. [9,10] reported a biosensor, gas sensor, and ion sensor using the MOSFET-BJT hybrid mode, which can be controlled by bias voltage to optimize each device. This sensor has high sensitivity and resolution compared to previous sensors and uses an innovative method. However, these methods are very expensive and require complex measurement system setups; for example, the fabrication process uses molecular beam, electrodeposition, photolithography, and thermal deposition [11]. Recently, the screening limitations of the field-effect transistor devices were revised and it was concluded that the detection of biomolecules in bio-samples was limited by ionic screening effects [12]. Moreover, a microfluidic biosensor was developed, but the fabrication procedure of this sensor relies on complex processes, such as photolithography [13].

To overcome these limitations and enhance sensitivity, a nanostructure optical biosensor was proposed. As described above, the nanostructure-based sensor has many advantages compared to other sensors. Its relatively large surface area and aspect ratio provide large binding sites. Yeom et al. [14] reported an anodic aluminum oxide (AAO)-based biosensor with high resolution and sensitivity compared to previous SPR-based biosensors. This sensor is constructed using an electrochemical process, which is much simpler compared to other fabrication processes. Additionally, this sensor offers rapid response, high accuracy, and non-chemical labeling. However, the sensor chip was prepared using mild anodization (MA) and, thus, the AAO chip fabrication time was very long. Despite these limitations, this type of sensor has been widely studied. Using this sensor, fabricated nano-porous AAO forms a sine wave through the interference of light, enabling observation of the peak point shift of the reflection wave according to antigen-antibody reaction. Additionally, the magnitude of this reflected wave can be increased through localized SPR (LSPR), which can confine the light in the nanostructure, enabling a family of plasmonic biosensing techniques [3,4,15]. LSPR biosensing techniques comprise refractive index (RI) biosensing, metal surface-enhance fluorescence (SEF), and surface-enhanced Raman scattering (SERS), all of which have the advantage of being label-free and highly sensitive compared to the conventional biosensors. Among these techniques, RI biosensing is the simplest, because the changes in the bulk RIs can be monitored by the shifts in the position or intensity of LSPR peaks with real-time and label-free sensing capability. Moreover, RI biosensing requires aluminum (Al) which, compared to conventional planar metals, such as Au and Ag, is inexpensive, naturally abundant, and is highly stable.

In this study, we improved the sensitivity of the AAO chip by pore-area control and reduced the manufacturing time of the device compared with a previous report using hard anodization (HA). The anodizing time was reduced to by approximately 10-fold to form an aluminum oxide layer of the same thickness, provided higher AAO chip production efficiency, and reduced the unit cost of the AAO chip [16]. We optimized the pore-area of AAO chip by controlling the fabrication parameters (applied voltage, electrolyte, anodizing time) to enhance the sensitivity of the optical biosensor. The measured limit of detection for the serum amyloid A1 (SAA1) antigen was 11.8 ag/mL. SAA1 is a member of a family of proteins synthesized in a multigene complex and is a well-known acute-phase reactant. Additionally, SAA1 is considered the most sensitive protein indicating inflammatory activity [17] and is a lung and gastric cancer biomarker [18,19] that is difficult to detect using previously-described measurement techniques and, thus, secondary data analysis is typically required.

## 2. Principle of the Proposed Biosensor 

**Figure 1:**
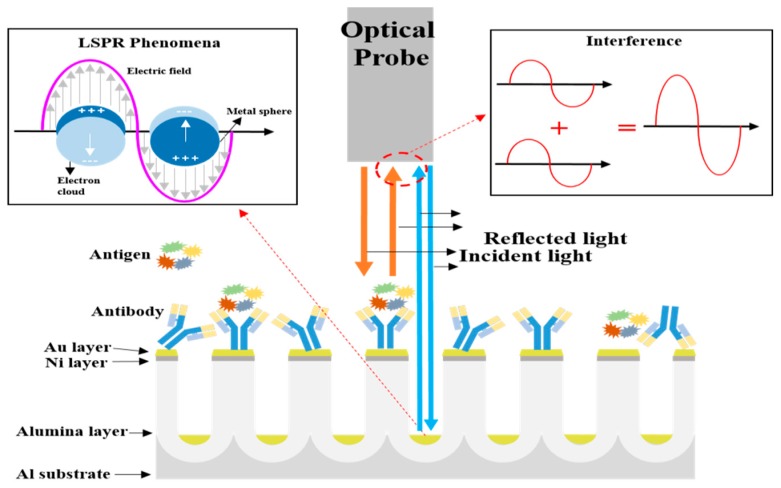
Schematic diagrams representing localized surface plasmon resonance and optical interferometry in nano-porous nanostructures.

In this study, we used an optical biosensor chip that can detect the SAA1 antigen using optical interferometry and LSPR occurring in the porous AAO layer. Figure 1 shows a schematic diagram of the LSPR phenomena and optical interferometry on the antigen-antibody-conjugated AAO chip [14,20,21,22].

As shown in Figure 1, optical interferometry occurred in front of the optical detector because the two lights (reflected light from antigen-antibody-conjugated Au sheet and reflected light from the bottom of Al substrate) meet and form the interference wave by interacting with constructive interference and destructive interference [23,24]. Additionally, the target substance was detected by measuring the changes in the reflected wavelength formed by changes in the effective optical thickness of the surrounding media. Rossi [23] described the effective optical thickness as the product of the refractive index and thickness of surrounding media. LSPR occurs when light interacts with a particle that is much smaller than the incident wavelength, leading to a plasmon that oscillates locally around the nanoparticles with a specific frequency. To explain the relationship between LSPR and interferometry more clearly, we used the model described below, which relies on the refractive index response of the propagating surface plasmon on a metal surface, as shown by Equation (1) [25]:

(1) $$\Delta \lambda_{\max}=m\Delta n[1-e^{\left(-2d/l_{d}\right)}]$$

In Equation (1), Δ*λ*_max_ is the wavelength shift, *m* is the refractive index sensitivity, Δ*n* is the change in refractive index induced by adsorption, *d* is the effective thickness of the layer, and *I_d_* is the characteristic length of the electromagnetic-field decay. The changes in refractive index and effective optical thickness of the surrounding media were induced when antigen-antibody binding occurred on the surface of the AAO chip. According to Equation (1), LSPR can measure small changes in the interfacial refractive index of the AAO surface.

## 3. Materials and Methods

### 3.1. Fabrication of the Surface-Area-Controlled AAO Nano-Structure

The purchased Al sheet for anodic aluminum oxide membrane production was highly pure (99.999%), but the Al sheet had a very thin native oxide layer that acts as a protective layer and was not uniform and non-porous. Consequently, we removed this layer and formed a uniform nano-porous oxide layer using a second anodizing method instead of a first anodizing method [26,27]. In the second anodizing method, which included the electrochemical oxidation process and can generate a uniform self-organized porous nano-structure, Al acts as the anode and the carbon plate acts as the cathode in the electrolyte solution [26,27]. The pore size of the oxide layer was controlled by varying the anodizing conditions (e.g., applied voltage, solution temperature, and concentration of acids, etc.). We also used a hard anodization (HA) method rather than the previously-reported mild anodization (MA) method for simple and fast fabrication of a highly-ordered AAO nano-structure [16]. The AAO nanostructure was fabricated as follows. The Al sheet (99.999%, 0.5 mm thickness Al, Goodfellow, Huntingdon, England) was used as the substrate, and phosphoric acid (3 M) was used as the electrolyte solution. The anodizing temperature was set to 9 °C. First, the Al sheet was ultra-sonicated in deionized water to remove dust. Next, 20 V was applied to remove the native oxide layer and other impurities on the surface in a solution of ethanol (30%) and perchloric acid (70%). Various voltages were applied for 30 min at 9 °C in electrolyte solution to fabricate the AAO. During this process, we gradually increased the voltage to the desired voltage by 1 V/s for using HA. HA is often accompanied by a large amount of heat due to high current generated through the electric field at the oxide-metal interface. The heat can partially damage the pore structure. Thus, to prevent this, a chiller was used to continuously circulate water at a low temperature to control the heat generated. After the first anodization, the oxide of Al sheet was removed by placing the sheet into stripping solution containing phosphoric acid (6 wt %) and chromic acid (1.8 wt %) for 90 min at 60 °C. The stripped Al sheet was re-anodized in a phosphoric acid solution using the same process as in the first anodization. The second anodizing time varied with applied voltage because the anodizing condition to form a 1-μm-thick AAO was different. The uniformity of the pore depends largely on the pre-patterned Al foil; however, the effect of the pore’s thickness on the sensitivity of the sensor is not negligible [14]. For devices with a thickness of less than 1 µm, the uniformity of the pore is reduced, resulting in a rougher surface, thereby making the immobilization of the antibody on the non-pore area difficult. Furthermore, for a device with a pore thickness ≥1 µm, while the pore uniformity might increase with an increase in pore thickness, the device’s sensitivity tends to decrease because the effective optical thickness decreases depending on pore thickness. Therefore, we set the pore thickness at 1 µm and the fabrication conditions are shown in Table 1. Compared with the anodizing time used to fabricate previously-reported devices, the anodizing time was greatly reduced by using the HA method.

### 3.2. Sensing Membrane Preparation

To immobilize the antibody on the AAO, a 5-nm-thick Ni and a 15-nm Au layer were deposited on the anodized AAO using an electron-beam evaporator. The average deposition rate of the evaporator was set to 0.1 Å/s under a vacuum of 4.5 × 10^−6^ T. The Ni layer can make the morphology of the Au layer uniform and enhance the plasmon resonance of the Au layer, which plays a pivotal role in LSPR and antibody immobilization [28]. To form the 11-mercaptoundecanoic acid self-assembly monolayer (SAM), thiol possessing carboxylic terminal groups on the Au-coated surface (the Ni- and Au-deposited porous Al sheet) was soaked in a solution of 11-mercapto-1-undecanol (20 mM) in ethanol for 24 h [29,30]. To activate the carboxylic groups formed on the surface, the solution comprising *N*-hydroxysuccinimide (50 mM) and *N*-(3-dimethylaminopropyl)-*N*-ethylcarbodiimide hydrochloride (50 mM) are reacted for 1 h to form amino groups at the terminal functional groups. The antibodies are then immobilized via amino coupling. Finally, by injecting the SAA1 antibody (60 μg/mL), we immobilized the SAA1 antibody at room temperature for 1 h and rinsed the sensing membrane by Dulbecco’s phosphate-buffered saline (DPBS) to remove the unbound antibody. The antigen and antibody of SAA1 were obtained from Protan Bio Co., Ltd. (Seoul, Korea) and Dulbecco’s phosphate-buffered saline, *N*-(3-dimethylaminopropyl)-*N*-ethylcarbodiimide hydrochloride, *N*-hydroxysuccinimide, and ethanol were obtained from Sigma Aldrich (St. Louis, MO, USA).

### 3.3. Sensing System for the SAA1 Detection Setup

**Figure 2:**
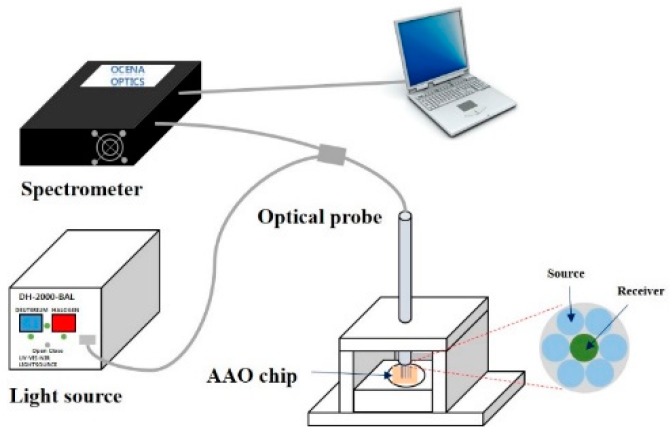
Schematic diagram of used sensing system to measure the SAA1 antigen concentration.

Figure 2 shows the optical measurement system comprised a white-light source (DH-2000-BAL, Ocean Optics Inc., Dunedin, FL, USA), reflectance optical probe (QD-400, Ocean Optics, Inc.), antigen-antibody reaction chamber made of Teflon, and a spectrometer (QE65000, Ocean Optics, Inc.). The spectrometer, which could measure absorbance and reflectance, was operated over a wide wavelength (200–1100 nm) with high resolution (0.17 nm). During the sensing process, we measure the reflected light from the fabricated Al sheet that had undergone an antigen-antibody reaction. As shown in Figure 2, the perpendicularly-irradiated light on the AAO chip was reflected from the AAO chip and the reflected light was collected through the same optical probe that had one receiver in the center and six sources surrounding the receiver. The collected light was analyzed with spectrum analysis software (spectrasuit, Ocean Optics, Inc.) in real-time.

## 4. Results and Discussion

### 4.1. Surface Characteristics of the Fabricated AAO Chip

**Figure 3:**
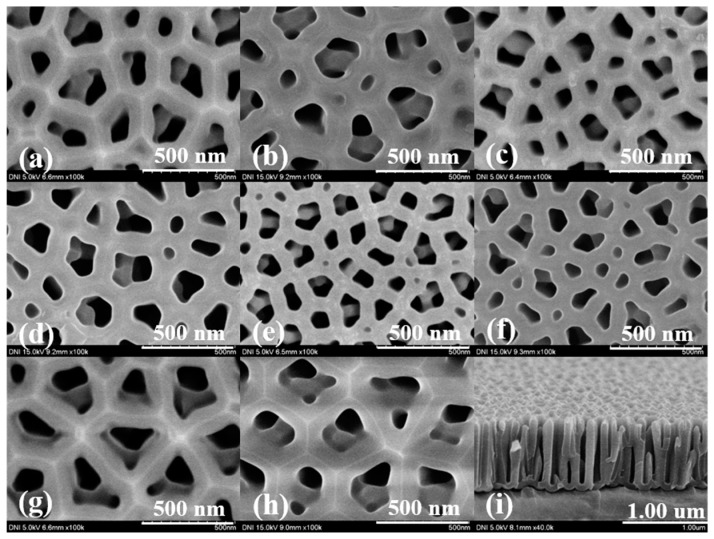
SEM image of AAO nanostructure: Top view SEM image of AAO nanostructure fabricated by (**a**) 60 V, (**b**) 70 V, (**c**) 80 V, (**d**) 90 V, (**e**) 100 V, (**f**) 110 V, (**g**) 120 V, (**h**) 130 V, and cross-sectional view (**i**).

The AAO chip with various pore sizes ranging from 117 nm to 267 nm was prepared using the second anodizing method and HA by varying the applied voltage and anodizing time. The surface characteristics of the fabricated AAO nanostructure were analyzed by field emission scanning electron microscopy. The results are shown in Figure 3 and Table 2, which describe the pore size, percentage of pore area, and percentage of non-pore area. Each value (pore size, and pore, and non-pore areas) was calculated using five SEM images taken from different regions of the device’s surface to ensure the reliability, and the average value and standard deviations were also calculated. To obtain the pore and the non-pore area ratio, all areas corresponding to the pores in the SEM were sampled and added. Next, the ratio of the pore-area was determined as the ratio of the sum of the area of the sampled pore and total area of the SEM image (dividing the sum of the area of the sampled pore by the total area of the SEM image, and multiplying by 100). The non-pore area was calculated using previously obtained values. As shown in Figure 3, the pore size differed, but the size of the unit cell increased. Additionally, the non-pore area increased as applied voltage and pore size increased. When a high voltage was applied, more oxidation occurred on the aluminum surface. However, this did not only increase the pore size, but also increased the unit cell in the aluminum oxide layer, resulting in a wider non-pore area [31]. There was no significant difference in devices fabricated at voltages greater than 130 V except for the oxidation time for forming the 1-μm-thick aluminum oxide layer. This non-pore area is important because this value is related to the probability of antibody immobilization; for a larger non-pore area, the probability of antibody immobilization on the AAO chip is increased because of the greater number of binding sites available for antibody immobilization.

### 4.2. Optical Properties of the Fabricated AAO Chip

**Figure 4:**
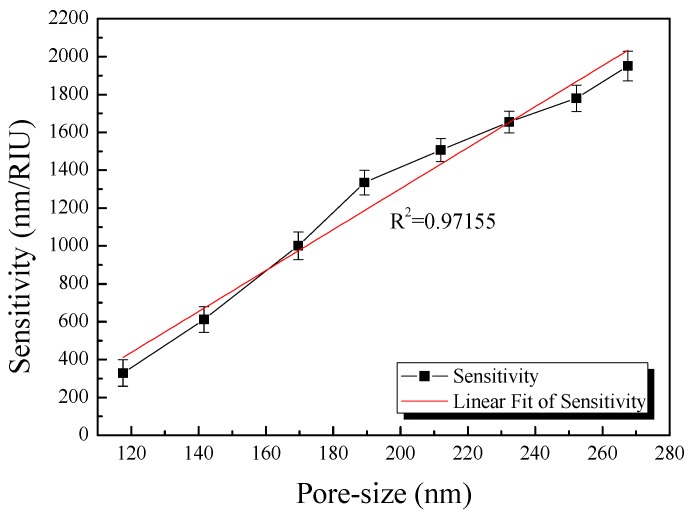
Average wavelength shift per refractive index unit (RIU) according to pore size.

To measure the optical sensitivity of the fabricated device, the surface of the devices was coated with various concentration of glycerin (3, 6, 9, 12, 15 wt %), which is a refractive index solution, and the reflected wavelength shift was measured. The refractive indices of the glycerin solutions were 1.3359, 1.3392, 1.3430, 1.3458, and 1.3492, respectively, which were measured using an Abbe refractometer. Glycerin thin films were formed by spin-coating and the average wavelength shifts according to refractive index unit were calculated and are shown in Figure 4.

As shown in Figure 4, the pore size and optical sensitivity were confirmed to be highly linear (R^2^ = 0.97155), confirming that optical sensitivity increased as pore size increased [32,33]. This sensitivity difference is proportional to the amount of glycerin deposited on the device surface. Glycerin deposited on the device surface changes the refractive index of the device surface. This constant value change affects the LSPR phenomena and causes a difference in sensitivity. These results indicate that as the non-pore area of the device increased, the surface area that could bind to the substance also increased, indicating that a greater amount of substance can be captured on the surface of the device and cause larger refractive index variations in the surrounding media, as explained by Equation (1).

### 4.3. Optimization of the AAO Fabrication Conditions

**Figure 5:**
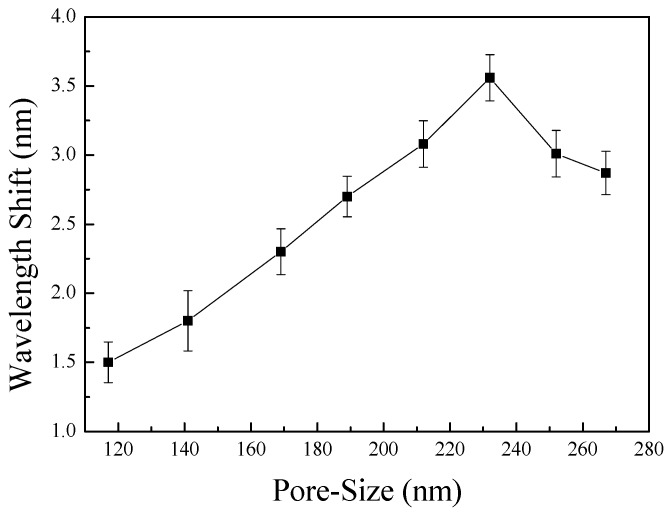
Average wavelength shifts and error bars according to pore size (117 nm, 141 nm, 169 nm, 189 nm, 212 nm, 232 nm, 252 nm, 267 nm) with 60 μg/mL of the antibody.

Due to the repulsive force and aggregation between the antibodies, the probability of antibody immobilization is not proportional to the non-pore area [34]. We identified the optimal area and fabrication conditions under which the repulsive force and aggregation of the antibody were minimized. An antibody at a concentration of 60 μg/mL was reacted on the surface of the SAM-treated device for each device and the wavelength shift was measured (Figure 5). This experiment was conducted in triplicate.

As shown in Figure 5, the results revealed partial linearity between the applied voltage and wavelength shift in the 60–110 V range, but generally decreased after reaching the maximum value of 3.5 nm at 110 V and then decreased again. As described above, as the non-pore area increased, the immobilization probability of the antibody increased, resulting in larger wavelength shifts in the device over 60–110 V. However, because of the repulsive force and aggregation between antibodies, such wavelength shifts are no longer increased and are decreased over a certain area (over 120 V) [34]. As the number of antibodies that could not bind to the surface of the device increased, the change in refractive index at the surface of the device was reduced and the wavelength change decreased. Based on these results, the optimal fabrication conditions of the device is 110 V.

### 4.4. Response Properties of the Optical Biosensor

**Figure 6:**
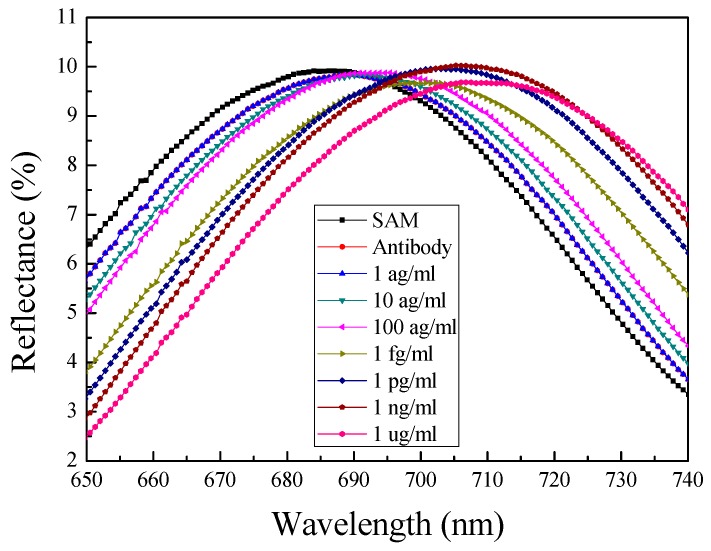
Measured reflection spectrum when various concentrations of SAA1 antigen (1 ag/mL, 10 ag/mL, 100 ag/mL, 1 fg/mL, 1 pg/mL, 1 ng/mL, 1 μg/mL) were injected with the SAA1 antibody immobilized.

**Figure 7:**
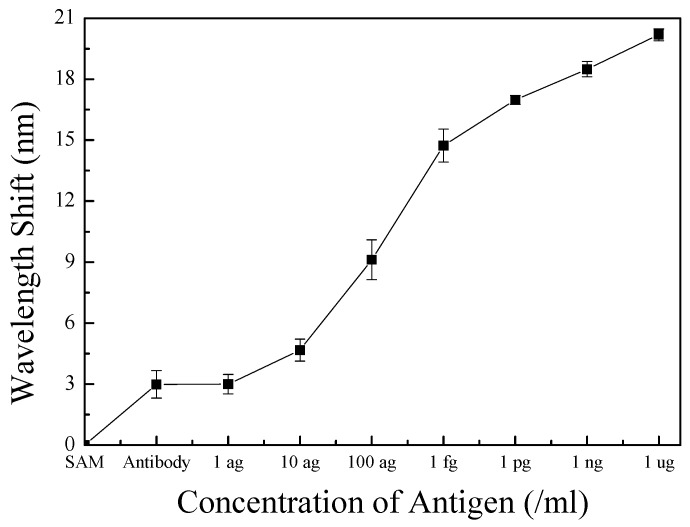
Average peak point shifts and error bars of the measured reflection wavelength according to various injected SAA1 antigen concentrations (1 ag/mL, 10 ag/mL, 100 ag/mL, 1 fg/mL, 1 pg/mL, 1 ng/mL, 1 μg/mL).

Finally, the bio-materials were reacted to confirm the application potential of the fabricated sensor as a biosensor using the lung cancer diagnosis biomarker SAA1 [18]. The SAA1 antigen/antibody used in this study was diluted in DPBS and injected at various concentrations (1 ag/mL, 10 ag/mL, 100 ag/mL, 1 fg/mL, 1 pg/mL, 1 ng/mL, and 1 µg/mL) and the reflected wavelength was subsequently measured (Figure 6). After antigen injection at each varying concentrations, the device was washed using DPBS to remove any antigens that might have physically adsorbed onto the surface of the device or any antibodies, other than those fixed on the surface of the device, by specific chemical bonding. This ensured that the measured wavelength shift occurred only due to an antigen-antibody reaction, specifically bound to the surface of the device, and not due to the antigen being physically adsorbed onto the surface of the device or antibody. The measured reflected wavelength represents the sine waveform, which results from interference between the light reflected from the inside of the pore and light reflected from the top of the pore [14]. The concentration of the antibody used for immobilization was 60 μg/mL and the peak of the reflection wavelength after antibody binding moved by approximately 3 nm. The wavelength shifts of the peak point according to the antigen concentration were as follows: 10 ag/mL (4.67 nm), 100 ag/mL (9.11 nm), 1 fg/mL (14.73 nm), 1 pg/mL (16.98 nm), 1 ng/mL (18.495 nm), and 1 μg/mL (20.18 nm). Such a shift in wavelength is caused by antigen-antibody binding on the surface of the sensing membrane. As the concentration of the injected antigen increased, the number of antigen-antibody bonds formed on the surface the device increased. As a result, the refractive index of the device surface showed a greater change, which was amplified by LSPR, resulting in a larger wavelength change. This phenomenon is explained by Equation (1). The Δ*n* caused by antigen-antibody binding changed the reflected wavelength, and as the value of Δ*n* increased, the wavelength change also increased. To verify the reliability of the device, five iterative experiments were carried out and the average peak points and error bars of the measured wavelengths are shown in Figure 7.

However, as shown in Figure 6 and Figure 7, there was no visible shift in the wavelength with an antigen concentration of 1 ag/mL. The sensitivity improved with an increase in the amount of SAA1 antibodies immobilized on the surface; however, an antigen concentration of 1 ag/mL was too low to be captured device. However, a shift in wavelength was observed when 10 ag/mL of the antigen was injected. Furthermore, as shown in Figure 7, the measured results represent two linear segments. First, the wavelength changes per the concentration of the injected antigen, and the interval spanning 10 ag/mL to 1 fg/mL shows a linear section, where the concentration of the injected antigen increases by 10-fold. Second, a partially linear section appears from 1 fg/mL to 1 µg/mL, where the concentration of the injected antigen increases by 1000-fold. This demonstrates that that the device is sensitive to antigens at low concentrations. This confirmed that the shift in wavelength was dependent on the SAA1 antigen concentration, and the measured limit of detection (LOD) was 11.8 ag/mL. In the process of calculating the LOD, we used the analytical method. Since the measured results show linearity at low concentration, we can obtain the slope (b) and standard deviation of the y-intercepts (Sa) by linear regression, and the LOD was calculated as three times the Sa divided by b [35].

**Figure 8:**
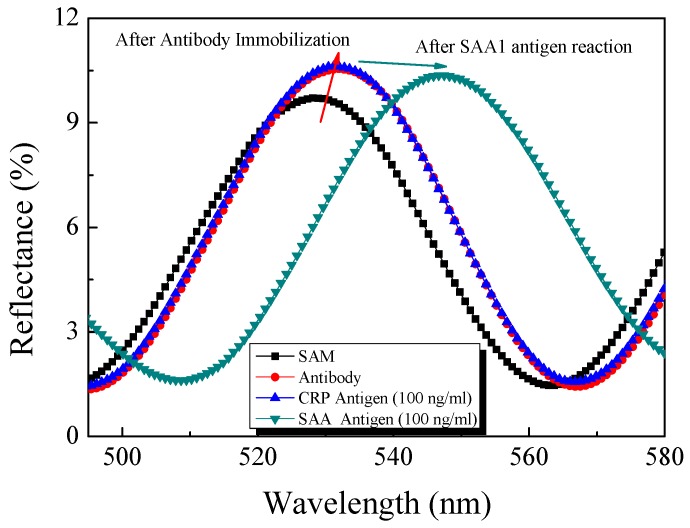
Wavelength shift according to concentration of antigen: SAM-treated AAO chip (black line); 60 μg/mL SAA1 antibody immobilized on the SAM-treated AAO chip (red line); injected 100 ng/mL CRP antigen (blue line); and 100 ng/mL SAA1 antigen (green line).

To confirm the selectivity of the fabricated biosensor chip, C-reactive protein (CRP), an inflammatory protein, was reacted on the surface of the SAA antibody-immobilized device [36]. The concentration of the injected CRP antigen was 100 ng/mL and the measured reflection wavelength is shown in Figure 8. A larger concentration of the CRP antigen was injected compared to SAA antigen, but there was no change in the measured reflected wavelength. Thereafter, when the same concentration of SAA antigen was injected again, a wavelength change of approximately 19 nm was observed, as previously confirmed. The CRP antigen, which is a heterogeneous substance, cannot bind to the surface of the SAA1 antibody-immobilized device and the CRP antigen that is not bound is washed out during the washing process. Thus, this system exhibited high selectivity and did not bind to heterologous materials.

## 5. Conclusions

In this study, we proposed a pore size/pore area-controlled optical biosensor based on the AAO nanostructure. As the pore size of the AAO chip increased, the non-pore area of the device increased. As a result, the number of antibodies capable of binding to the device surface was increased. This allowed for a larger number of antibodies to be immobilized on the surface of the device and, thereby, a smaller amount of antigen could be detected. The sensitivity of the fabricated biosensor was 11.8 ag/mL, determined by measuring the wavelength shift according to the refractive index change of the surrounding media, which confirmed that the sensitivity of the biosensor increased with increasing pore size and non-pore area. A reaction with the SAA1 antigen, an inflammatory protein, confirmed that the fabricated device was highly sensitive to the SAA1 antigen, as the number of antibodies increased because of the increase in the non-pore area. The device did not bind heterologous CRP antigen, confirming that the proposed optical sensor was highly selective for the SAA1 antigen. Additionally, by using HA, the anodizing time was reduced, thereby reducing the overall device fabrication time and simplifying device fabrication. This proposed optical biosensor can be used to detect small-molecule, enzymes, and other materials that require high sensitivity and selectivity, are present at a low concentration, or are small in size.

## Figures and Tables

**Table 1:** Second anodizing time according to the applied voltage in the anodizing process to fabricate a porous 1-μm-thick oxide.

Applied Voltage (V)	2nd Anodizing Time (min)
60	25
70	20
80	18
90	15
100	11
110	8.5
120	6
130	4

**Table 2:** Pore size and percentage of pore area and non-pore area.

Applied Voltage (V)	Pore Size (nm)	Percentage of Pore Area (%)	Percentage of Non-Pore Area (%)
60	117 ± 2.967	30.98	69.02 ± 0.545
70	141 ± 2.521	27.78	72.22 ± 0.671
80	169 ± 2.942	25.62	74.38 ± 0.367
90	189 ± 3.247	21.60	78.40 ± 0.841
100	212 ± 3.156	19.61	80.39 ± 0.541
110	232 ± 2.782	17.16	82.84 ± 0.412
120	252 ± 2.897	13.67	86.33 ± 0.352
130	267 ± 2.564	11.44	88.56 ± 0.415
